# A Positive Relationship between Betel Nut Chewing and Significant Liver Fibrosis in NAFLD Subjects, but Not in Non-NAFLD Ones

**DOI:** 10.3390/nu13030914

**Published:** 2021-03-11

**Authors:** Yu-Tsung Chou, Chung-Hao Li, Zih-Jie Sun, Wei-Chen Shen, Yi-Ching Yang, Feng-Hwa Lu, Chih-Jen Chang, Jin-Shang Wu

**Affiliations:** 1 Department of Health Management Center, National Cheng Kung University Hospital, College of Medicine, National Cheng Kung University, Tainan 70403, Taiwan; odyssey4324@gmail.com (Y.-T.C.); smallhear@gmail.com (C.-H.L.); 2 Department of Family Medicine, National Cheng Kung University Hospital, College of Medicine, National Cheng Kung University, Tainan 70403, Taiwan; sunzihjie@gmail.com (Z.-J.S.); ab-genius@hotmail.com (W.-C.S.); yiching@mail.ncku.edu.tw (Y.-C.Y.); fhlu@mail.ncku.edu.tw (F.-H.L.); 3 Department of Family Medicine, College of Medicine, National Cheng Kung University, Tainan 70101, Taiwan; 4 Department of Family Medicine, National Cheng Kung University Hospital Dou-Liou Branch, College of Medicine, National Cheng Kung University, Yunlin 64043, Taiwan; 5 Department of Family Medicine, Ditmanson Medical Foundation Chia-Yi Christian Hospital, Chiayi 60002, Taiwan

**Keywords:** betel nut, *Areca catechu*, NAFLD, nonalcoholic fatty liver disease, liver fibrosis

## Abstract

Background: Betel nut chewing is associated with oral cancer, cardiovascular disease, liver cirrhosis, and hepatocellular carcinoma (HCC). The aim of this study was to explore the association of betel nut chewing with liver fibrosis in subjects with and without nonalcoholic fatty liver disease (NAFLD). Method: A total of 5967 subjects were enrolled. NAFLD was diagnosed with ultrasonography. Betel nut chewing was classified into non-chewing, ex-chewing, and current chewing, and cumulative dosages were calculated. The aspartate aminotransferase (AST)/platelet ratio index and NAFLD fibrosis scores (NFS) were calculated for evaluation of liver fibrosis. Results: NAFLD increased the associated risk of liver fibrosis in those with (odds ratio (OR): 5.51, 95% confidence interval (CI): 3.09–9.80) and without betel nut chewing (OR: 2.33, 95% CI: 1.64–3.29). In subjects without NAFLD, betel nut chewing was not associated with liver fibrosis (OR: 1.12, 95% CI: 0.44–2.86). In subjects with NAFLD, cumulative betel nut chewing and ex- and current chewing were positively associated with NFS and significant liver fibrosis. Conclusions: In subjects with NAFLD, betel nut chewing, even ex-chewing, was associated with a higher risk of liver fibrosis, where higher cumulative levels were found to increase the risk of significant liver fibrosis. However, the associated risk of liver fibrosis due to betel nut chewing was insignificant in subjects without NAFLD.

## 1. Introduction

Betel nut is the seed of *Areca catechu*, which grows in tropical areas, and betel nut chewing is common in tropical Asia [1]. As increasing numbers of Asians have immigrated to Western countries, increased betel nut use has also been observed in Western countries such as the United Kingdom, the United States, and Australia [2]. It is estimated that about 600 million people use betel nut worldwide to engage in social customs, religious practices, and cultural rituals, or as a psychoactive substance [1,3]. In Taiwan, betel nuts are often chewed with leaves, flowers, or stems of *Piper betle*, or chewed with additives such as tobacco, cardamom, catechu, slaked lime, or cloves [4,5]. Betel nut chewing is associated with a risk of oral cancer due to the carcinogenic effect of arecoline, polyphenols, and tannins within betel nuts, as well as due to safroles in *Piper betle* leaves, flowers, and stems [6]. Therefore, betel nut is classified as a group 1 carcinogen by the International Agency for Research on Cancer [3,7]. Epidemiological studies have also demonstrated that betel nut chewing is associated with an increased risk of obesity and metabolic syndrome, hypertension, type II diabetes, cardiovascular disease, and even all-cause mortality [8,9].

Nonalcoholic fatty liver disease (NAFLD) is currently one of the most common liver diseases [10,11]. It has been found to be related to obesity, hypertension, dyslipidemia, diabetes mellitus, cardiovascular diseases, and even mortality [12]. In addition, NAFLD also increases the risk of liver fibrosis, liver cirrhosis, and hepatocellular carcinoma [13,14]. Liver fibrosis has been shown to be the strongest predictor of disease-specific mortality in NAFLD [15,16]. However, the diagnosis of liver fibrosis, liver biopsy, despite being the gold standard for clinical assessment, not only is costly but also has a risk of severe complications [17]. Several scoring systems incorporating clinical parameters and laboratory tests have been designed for noninvasive evaluation of liver fibrosis. Some scoring systems, such as the aspartate aminotransferase (AST)/platelet ratio index (APRI) and the NAFLD fibrosis score (NFS), are commonly used in clinical practice [17,18]. For evaluation of liver fibrosis in patients with NAFLD, for example, the NFS has been validated and is recommended by Western society [19,20].

In terms of the relationship between betel nut chewing and liver diseases, previous studies demonstrated that betel nut chewing is associated with liver cirrhosis and hepatocellular carcinoma [21,22]. Nonalcoholic fatty liver disease (NAFLD) is one risk factor for liver fibrosis, cirrhosis, and hepatocellular carcinoma. However, studies on the relationship between betel nut and liver fibrosis taking NAFLD into consideration are lacking, and there is only one case report revealing an association between nonalcoholic steatohepatitis (NASH) and betel nut chewing [23]. Therefore, this study was conducted to investigate the association between betel nut chewing and liver fibrosis by evaluating APRI and NFS in subjects with and without NAFLD.

## 2. Methods

### 2.1. Study Population

The study population was derived from participants who visited the health examination center for self-motivated physical checkups at National Cheng Kung University Hospital (NCKUH), from October 2006 to August 2009. An analysis for secondary data without any personally identifiable information was performed, and the study protocol was approved by NCKUH’s Institutional Review Board (IRB Number: A-ER-107-285) in Tainan, Taiwan. A total of 5967 subjects were included in this study after excluding those aged <18 years, those with a history of cancer, hepatitis B, hepatitis C, or other cause of chronic liver disease (such as autoimmune and drug-related liver disease), those taking medications for metabolic derangements, including diabetes, hypertension, dyslipidemia, and hyperuricemia, those engaging in heavy alcohol consumption (subjects with weekly alcohol consumption ≥140 g) [24,25,26], and those with incomplete data.

### 2.2. Measures

Each participant’s baseline data were collected, including personal demographic information, medical history, medication use, and lifestyle habits, such as betel nut chewing, cigarette smoking, alcohol consumption, and exercise. Betel nut chewers were categorized into non-chewers, ex-chewers, and current chewers. Participants who chewed betel nut at least once weekly in the previous 6 months were defined as current chewers [5]. Ex-chewers were defined participants who previously chewed betel nut at least once per week for no less than 6 months but ceased for at least the last six months. Duration (years) and quantity (pieces/day) of betel nut use were also collected, and cumulative exposure to betel nut was also calculated on the basis of the quantity per day in piece × duration in years. Cumulative smoking was evaluated in terms of pack-year multiplied by number of years each person smoked and the average daily smoking amount (pack/day). Average alcohol consumption, measured as grams per week, was determined as the product of the amount of alcohol consumed and the alcohol drinking frequency by each person. Regular exercise was defined as vigorous exercise for a minimum of 20 min at least three times weekly.

Body mass index (BMI) was defined as weight (kg)/square of height (m^2^), and obesity was defined as BMI ≥ 27kg/m^2^ [27]. Right brachial systolic and diastolic blood pressures (BP) were measured for each participant in the supine position after resting for at least 10 min. Hypertension was defined as systolic BP ≥ 140 mmHg, diastolic BP ≥ 90 mmHg, or a medical history of hypertension. The laboratory data included fasting plasma glucose (FPG), 2 h post-load glucose, glycated hemoglobin (HbA1c), total cholesterol (TC), triglyceride (TG), high-density lipoprotein cholesterol (HDL-C), albumin, alanine aminotransferase (ALT), aspartate aminotransferase (AST), uric acid, and platelets. Diabetes mellitus was defined as an FPG level ≥ 126 mg/dL, a 2 h post-load glucose level ≥ 200 mg/dL, HbA1c ≥ 6.5%, or a past history of diabetes [28]. Hyperuricemia was defined as serum uric acid level ≥ 7 mg/dL in males and ≥ 6 mg/dL in females, respectively [29]. To verify liver fibrosis, APRI (the aspartate aminotransferase/platelet ratio index) [30] was calculated for all subjects. In the subjects with NAFLD, NFS is commonly used for a better evaluation of liver fibrosis [19,20]. The cutoff value for defining significant liver fibrosis was APRI ≥ 0.5 for total subjects and NFS ≥ −1.455 for subjects with NAFLD [31].

Abdominal sonography was performed and interpreted by experienced radiologists with high-resolution ultrasonography (Xario SSA-660A; Toshiba, Nasu, Japan) using a 3.5 MHz linear transducer. The diagnosis of NAFLD was made on the basis of the following sonography findings: (1) decrease in lucidity or poor visualization of the borders of the diaphragm and intrahepatic vessel walls; (2) increase in hepatic echogenicity; (3) attenuation of the penetrated ultrasound signal [32,33].

### 2.3. Statistical Analysis

SPSS software (v. 22.0, SPSS, Inc., Chicago, IL, USA) was used for the data analysis. Categorical and continuous variables were presented as numbers (percentages) and the means ± standard deviations, respectively. For comparisons of clinical variables between subjects with and without NAFLD, a Pearson’s chi-square analysis and an independent *t*-test were performed to provide comparisons of the categorical variables and continuous variables separately. In the multivariate analysis, a multiple logistic regression was initially used to explore the association of betel nut chewing and NAFLD with the risk of significant liver fibrosis (defined as APRI ≥ 0.5). Then, in the NAFLD subjects, the association of betel nut chewing habit (including different duration, amounts, and cumulative exposure) with NFS was investigated using a linear regression model. Lastly, a logistic regression model was applied to evaluate the relationship between betel nut chewing and significant liver fibrosis (defined as NFS ≥ −1.455). The adjustment variables included age, gender, obesity, diabetes mellitus, smoking, and alcohol consumption. Statistical significance was defined as *p* < 0.05 throughout the analyses. 

## 3. Results

Table 1 shows the comparisons of the clinical characteristics of the subjects with and without NAFLD. Among all 5967 subjects, 1915 (32.1%) participants were diagnosed with NAFLD. Subjects with NAFLD were significantly older and tended to be male, and they had higher systolic and diastolic blood pressure, BMI, AST, ALT, uric acid, APRI, and NFS, as well as more prevalent histories of hypertension, diabetes mellitus, hyperlipidemia, cumulative smoking, and average alcohol use. In addition, subjects with NAFLD had a higher prevalence of significant liver fibrosis (7.5% vs. 2.1%, *p* < 0.001), ex and current betel nut chewing (ex-chewing: 4.6% vs. 3.2%, *p* < 0.001; current chewing: 2.8% vs. 1.0%, *p* < 0.001), and cumulative betel nut exposure (11.9 ± 68.7 piece-year vs. 6.7 ± 62.3 piece-year, *p* < 0.001) as compared to subjects without NAFLD. 

In addition, among subjects with NAFLD, both APRI (0.42 vs. 0.29, *p* < 0.001) and NFS (−2.54 vs. −2.87, *p* = 0.002) were significantly higher among betel nut chewers than non-chewers. Furthermore, the prevalence of significant liver fibrosis was also significantly higher among betel nut chewers by evaluation of both APRI (16.9% vs. 7.1%, *p* < 0.001) and NFS (29.4% vs. 19.6%, *p* = 0.005) than non-chewers (data shown in the supplementary file, Table S1).

In total subjects, Table 2 reveals a higher associated risk of significant liver fibrosis in NAFLD groups with (crude odds ratio (OR): 9.67; 95% confidence interval (CI): 5.91–15.80, *p* < 0.001) and without betel nut chewing (crude OR: 3.64; 95% CI: 2.73–4.85, *p* < 0.001) in the logistic regression model. After adjustment for other variables, NAFLD with and without betel nut chewing remained an elevated risk for significant liver fibrosis (adjusted OR: 5.51, 95% CI: 3.09–9.80, *p* < 0.001; adjusted OR: 2.33, 95% CI: 1.64–3.29, *p* < 0.001). In contrast, the risk of liver fibrosis did not reach statistical significance in the betel nut chewers without NAFLD in either the univariate or the multivariate analysis using the logistic regression model. We further analyzed whether betel nut chewing increased associated risk of significant liver fibrosis in NAFLD subjects. With the reference of NAFLD subjects without betel nut chewing, NAFLD subjects with betel nut chewing also had a higher risk of significant liver fibrosis (adjusted OR: 2.37, 95% CI: 1.42–3.95, *p* = 0.001) (data shown in the supplementary file, Table S2).

We then conducted a multiple linear regression analysis for further evaluation of betel nut chewing and the NFS, as a continuous variable, in the NAFLD subjects (Table 3). Both ex and current betel nut chewing were found to be significantly related to the NFS (ex-chewing: B coefficient: 0.305, 95% CI: 0.112–0.497, *p* = 0.002; current chewing: B coefficient: 0.334, 95% CI: 0.093–0.574, *p* = 0.007). In addition, both small and large daily chewing amounts, cumulative exposure, and short durations of betel nut chewing were also related to a higher NFS. 

Table 4 demonstrates, in the presence of NAFLD, a positive association of ex and current betel nut chewing with significant liver fibrosis, as a dichotomous variable. The adjusted ORs were 2.41 (95% CI: 1.31–4.44, *p* = 0.005) in ex-chewers and 2.88 (95% CI: 1.35–6.15, *p* = 0.006) in current chewers. Furthermore, the risk of liver fibrosis was also significantly increased in NAFLD subjects who were chewing betel nut for a longer period of time, had a greater amount of daily betel nut consumption, and had a greater cumulative exposure. In addition, older age, obesity, and diabetes were positively related to significant liver fibrosis in subjects with NAFLD.

## 4. Discussion

The results indicated that betel nut chewing was associated with significant liver fibrosis in the subjects with NAFLD, but not in the subjects without NAFLD. To the best of our knowledge, this is the first study to investigate the association of betel nut chewing with significant liver fibrosis taking NAFLD into consideration. Previous studies discussing betel nut and liver diseases mainly focused on the relationship between betel nut chewing and liver fibrosis, liver cirrhosis, and HCC [9,21,22,34,35], especially in terms of the parallel relationship with the effects of hepatitis B virus (HBV) and hepatitis C virus (HCV) infection [34,35,36]. A study conducted by Hsiao et al. revealed that betel nut chewing is an independent risk factor for liver cirrhosis [22]. A study conducted by Tsai et al. also found an association between betel nut chewing and liver cirrhosis and HCC, along with an additive interaction between chronic HBV/HCV infection and betel nut chewing [21,35]. However, the role of NAFLD in betel nut chewing and liver fibrosis was not examined in these studies. There is only one case report revealing NASH (nonalcoholic fatty liver disease), as diagnosed with a liver biopsy, in one betel nut chewer [23], but the liver fibrosis status was not discussed in a larger sample [23]. We found that higher cumulative betel nut chewing increased the risk of significant liver fibrosis in subjects with NAFLD, although there was an insignificant association of betel nut chewing with liver fibrosis in the subjects without NAFLD. In addition, NAFLD subjects with betel nut chewing had a 2.37-fold higher risk of liver fibrosis than those without betel nut chewing after adjustment for other clinical variables.

The pathogenic mechanisms linking betel nut chewing, NAFLD, and the risk of liver fibrosis remain unclear. According to previous studies, betel nut chewing is related to metabolic disorders [8,9,37,38]. One meta-analysis concluded that betel nut chewing significantly increases risk of obesity, insulin resistance, metabolic syndrome, dyslipidemia, and diabetes [9], which are all common risk factors for developing NAFLD [39,40]. Under the current concept of the “multiple-parallel hits” hypothesis for progression of NAFLD [41,42], metabolic derangements caused by betel nut chewing appear to play a role in initiating the cascade of liver damage [43]. Furthermore, due to the effect of insulin resistance, glucotoxicity, and lipotoxicity, NASH occurs [44,45,46] as a more severe manifestation of NAFLD, resulting in not only fat deposition, but also hepatic tissue inflammation, cell damage, and subsequent fibrosis [39]. In this study, although obesity, hypertension, diabetes, dyslipidemia, and hyperuricemia were all adjusted in the multivariate analysis, there was still a positive association between betel nut chewing and liver fibrosis. Thus, further studies are needed to further clarify the relationship among betel nut chewing, NAFLD, and liver fibrosis.

The results of the present study also suggest that NAFLD is significantly related to liver fibrosis regardless of betel nut chewing habit, which is compatible with the current concept considering NAFLD as a risk factor associated with liver fibrosis [47]. However, in the current study, an insignificant relationship was found between betel nut chewing and liver fibrosis in the subjects without NAFLD. In the subjects without NAFLD, there was less fat accumulation in hepatic tissue, resulting in less potential for a lipotoxicity cascade induced by betel nut chewing. In addition, one hepatotoxic and carcinogenic effect of betel nut is caused by safrole, a compound in betel quid [48]. This reaction is mediated mainly by several types of cytochrome P450 (CYP450) isoenzymes [48]. Of all CYP isoenzymes involved in safrole metabolism, CYP2A6 (the most active CYP isoenzyme in generation of the toxic safrole metabolite) and CYP2C9 (the main enzyme of the CYP2C subfamily in the hepatic tissue) are much more bioactive in patients with NAFLD [49,50], which might partially result in an increased associated risk of liver fibrosis. Furthermore, NAFLD is associated with elevated reactive oxygen species (ROS) production, increased free radical levels, and increased oxidative stress, which result in hepatic inflammation and fibrogenesis. The presence of arecoline, a major alkaloid found in the betel nut [51], further deteriorates this inflammatory pathway by depressing the activities of antioxidants in hepatic tissue [51] and enhancing production of ROS [51,52,53] and inflammatory cytokines such as tumor necrosis factor (TNF)-alpha and transcription factors such as nuclear factor (NF)-κB [54,55,56]. On the contrary, in the absence of NAFLD, the levels of oxidative stress, inflammatory cytokines, and free radicals are all lower, and there is also less activity of CYP isoenzymes, resulting in a less favorable environment for the toxic effects of safrole and arecoline, which might provide some explanations for the insignificant relationship between betel nut chewing and liver fibrosis among the subjects without NAFLD.

In this study, liver fibrosis was also shown to be related to older age, male gender, BMI, dyslipidemia, and diabetes. These results are similar to those of previous studies [40,42,57,58,59]. As for age and liver fibrosis, it is well known that metabolic syndrome and insulin resistance are more prevalent in the elderly than in young people [60]. In addition, aging is related to increased ROS production, elevated oxidative stress, and poor response to hepatic tissue damage [60], which may be related to liver fibrosis. Females are less likely to develop liver fibrosis, which may be related to the effects of estrogen on the modulation of hepatic fibrogenesis [59]. In obese subjects, elevated free fatty acid levels, insulin resistance, visceral fat accumulation, and several proinflammatory mediators develop, which may result in hepatic steatosis, chronic hepatic inflammation, and subsequent liver fibrosis [57,61]. Diabetes also provokes liver fibrogenesis by elevating the production of leptin and TNF-α, which activates the inflammatory pathways that cause hepatic damage and fibrosis [62]. We also found an insignificant relationship between liver fibrosis and smoking, although previous studies showed cigarette smoking to be associated with liver fibrosis [63]. This relationship may be related to a healthy-worker-like effect, where it has been observed that workers typically exhibit lower overall death rates as compared to the general population because the severely ill and disabled are unable to work. In addition, smokers may change lifestyle when they suffer from diseases or are more aware of the harmful effects of smoking [64,65].

Despite the large sample size and relatively comprehensive serologic data with concomitant adjustment of important covariates of NAFLD, NASH, and liver fibrosis, there were also several limitations to this study. First, it was impossible to establish a causal relationship between betel nut chewing and liver fibrosis using a cross-sectional design. Second, since the population in this study was limited to subjects who received health examinations at a tertiary medical center, the results should be interpreted carefully when they are applied to the general population. Third, detailed dietary history was not available in this study. Fourth, the diagnosis of liver fibrosis was assessed by APRI and NFS, rather than other validated noninvasive evaluation methods such as Fibroscan [66]. Lastly, the diagnosis of NAFLD was done with an abdominal ultrasonographic examination, which may not be the gold standard for NAFLD diagnosis [67].

## 5. Conclusions

In conclusion, betel nut chewing, even ex-chewing, was found to be associated with a significant risk of liver fibrosis in subjects with NAFLD. Moreover, higher cumulative betel nut chewing was shown to increase the risk of significant liver fibrosis. On the contrary, in the subjects without NAFLD, the associated risk of liver fibrosis due to betel nut chewing was found to be insignificant.

## Figures and Tables

**Table 1:** Comparisons of participants’ clinical characteristics between subjects with and without nonalcoholic fatty liver disease (NAFLD).

Variables	NAFLD		*p*-Value
	No (*n* = 4052)	Yes (*n* = 1915)	
Age, years	45.1 ± 12.3	48.3 ± 11.1	<0.001
Male	1993 (49.2%)	1392 (72.7%)	<0.001
BMI, kg/m^2^	22.5 ± 2.6	26.7 ± 3.2	<0.001
SBP, mmHg	111.7 ± 15.3	121.3 ± 15.9	<0.001
DBP, mmHg	65.8 ± 9.8	72.4 ± 10.5	<0.001
FPG, mg/dL	88.2 ± 15.9	96.8 ± 24.6	<0.001
ALT, U/L	22.8 ± 13.7	40.1 ± 26.2	<0.001
AST, U/L	22.4 ± 8.9	28.1 ± 15.9	<0.001
Cholesterol, mg/dL	191.8 ± 35.8	205.5 ± 36.1	<0.001
Triglyceride, mg/dL	102.8 ± 58.8	169.1 ± 102.9	<0.001
HDL-C, mg/dL	53.2 ± 13.7	43.2 ± 10.2	<0.001
Cholesterol/HDL-C	3.8 ± 1.2	5.0 ± 1.3	<0.001
Creatinine, mg/dL	0.85 ± 0.41	0.91 ± 0.22	<0.001
Uric acid, mg/dL	5.6 ± 1.4	6.7 ± 1.5	<0.001
Hypertension	307 (7.6%)	353 (18.4%)	<0.001
Diabetes mellitus	185 (4.6%)	295 (15.4%)	<0.001
Alcohol consumption amount, g/week	12.0 ± 27.0	14.1 ± 25.8	<0.001
Cumulative exposure of smoking, pack-year	3.3 ± 9.4	5.9 ± 13.9	0.003
Exercise ≥3/week	492 (12.1%)	211 (11.1%)	0.209
Betel nut chewing, non-chewing	3882 (95.8%)	1772 (92.5%)	<0.001
Ex-chewing	129 (3.2%)	89 (4.6%)	
Current chewing	41 (1.0%)	54 (2.8%)	
Duration of betel nut use, years	0.40 ± 2.4	0.81 ± 3.6	<0.001
Duration of betel nut use, none	3882 (95.8%)	1772 (92.5%)	<0.001
≤10 years	123 (3.0%)	96 (5.0%)	
>10 years	47(1.2%)	47 (3.5%)	
Quantity of betel nut use per day	0.56 ± 3.5	0.94 ± 4.1	<0.001
Quantity of betel nut use, none	3882 (95.8%)	1772 (92.6%)	<0.001
≤5 pieces/day	39 (1.0%)	32 (1.7%)	
>5 pieces/day	131 (3.2%)	111 (5.8%)	
Cumulative exposure of betel nut, piece-year	6.7 ± 62.3	11.9 ± 68.7	<0.001
Cumulative exposure, none	3882 (95.8%)	1772 (92.6%)	<0.001
≤100 piece-year	85 (2.1%)	68 (3.6%)	
>100 piece-year	85 (2.1%)	75 (3.9%)	
APRI score	0.23 ± 0.15	0.29 ± 0.20	<0.001
APRI score ≥0.5	85 (2.1%)	150 (7.8%)	<0.001
NFS	−2.87 ± 1.18	−2.53 ± 1.25	<0.001
NFS ≥ −1.455	453 (11.3%)	389 (20.3%)	<0.001

Data are expressed as the mean ± standard deviation or number (percent). NAFLD: nonalcoholic fatty liver disease, BMI: body mass index, SBP: systolic blood pressure, DBP: diastolic blood pressure, FPG: fasting plasma glucose, ALT: alanine aminotransferase, AST: aspartate aminotransferase, HDL-C: high-density lipoprotein-cholesterol, eGFR: estimated glomerular filtration rate, APRI: AST/platelet ratio index, NFS: NAFLD fibrosis score. Normal reference values: FPG: <100 mg/dL, AST: male: 10–50 U/L, female: 10–35 U/L, ALT: male: <50 U/L, female: <35 U/L, cholesterol: <200 mg/dL, triglyceride: <150 mg/dL, HDL-C: male: >40 mg/dL, female: >50 mg/dL, LDL-C: <100 mg/dL, creatinine: male: 0.70–1.20 mg/dL, female: 0.50–0.90 mg/dL, uric acid: male: <7 mg/dL, female: < 6 mg/dL.

**Table 2:** Logistic regression model for risk of significant liver fibrosis (defined as APRI ≥0.5).

Variables	Univariate Analysis		Multivariate Analysis	
	OR (95% CI)	*p*-Value	OR (95% CI)	*p*-Value
NAFLD (−), Betel nut chewing (−)	Reference		Reference	
NAFLD (−), Betel nut chewing (+)	1.44 (0.58–3.60)	0.436	1.12 (0.44–2.86)	0.821
NAFLD (+), Betel nut chewing (−)	3.64 (2.73–4.85)	<0.001	2.33 (1.64–3.29)	<0.001
NAFLD (+), Betel nut chewing (+)	9.67 (5.91–15.80)	<0.001	5.51 (3.09–9.80)	<0.001
Age, 40–60 years vs. <40 years			1.13 (0.80–1.59)	0.492
Age, >60 years vs. <40 years			2.03 (1.32–3.13)	0.001
Male vs. female			1.58 (1.10–2.25)	0.013
BMI, kg/m^2^			1.05 (1.00–1.09)	0.048
Hypertension, yes vs. no			1.21 (0.85–1.72)	0.295
Diabetes, yes vs. no			1.80 (1.26–2.59)	0.001
Cholesterol/HDL-C ≥ 5, yes vs. no			1.01 (0.75–1.37)	0.938
Hyperuricemia, yes vs. no			1.18 (0.87–1.61)	0.307
Cumulative exposure of smoking, pack-year			1.00 (0.99–1.01)	0.658
Alcohol consumption amount, g/week			1.00 (1.00–1.01)	0.526

NAFLD: non-alcoholic fatty liver disease, APRI: AST/platelet ratio index, BMI: body mass index, HDL-C: high-density lipoprotein-cholesterol, OR: odds ratio, CI: confidence interval.

**Table 3:** Linear regression model for NAFLD fibrosis score and betel nut chewing among patients with NAFLD.

Variables	Model 1	Model 2	Model 3	Model 4
	B Coefficient (95% CI)	B Coefficient (95% CI)	B Coefficient (95% CI)	B Coefficient (95% CI)
Betel nut use, ex vs. non	0.305 (0.112–0.497) **			
Betel nut use, current vs. non	0.334 (0.093–0.574) **			
Quantity of betel nut use per day				
≤5 pieces/day vs. none		0.456 (0.152–0.761) **		
>5 pieces/day vs. none		0.272 (0.096–0.447) **		
Duration of betel nut use, years				
≤10 years vs. none			0.340 (0.156–0.523) **	
>10 years vs. none			0.260 (0.000–0.521)	
Cumulative exposure, piece-year ^a^				
≤100 piece-year vs. none				0.387 (0.173–0.600) ***
>100 piece-year vs. none				0.246 (0.036–0.455) *

All models adjusted for age, gender, body mass index, hypertension, diabetes, dyslipidemia, hyperuricemia, smoking (pack-year), and alcohol consumption (g/week). ^a^ Quantity per day in piece × duration in year. * *p* < 0.05, ** *p* < 0.01, *** *p* < 0.001.

**Table 4:** Logistic regression model for significant liver fibrosis (NFS ≥ −1.455) and betel nut chewing among patients with NAFLD.

Variables	Model 1	Model 2	Model 3	Model 4
	OR (95% CI)	OR (95% CI)	OR (95% CI)	OR (95% CI)
Age, 40–60 years vs. <40 years	11.64 (5.50–24.64) ***	11.54 (5.47–24.34) ***	11.44 (5.42–24.13) ***	11.48 (5.44–24.20) ***
Age, >60 years vs. <40 years	107.55 (48.41–238.94) ***	106.70 (48.17–236.37) ***	105.77 (47.75–234.27) ***	106.16 (47.93–235.12) ***
Male vs. female	0.99 (0.70–1.40)	0.99 (0.70–1.40)	0.99 (0.70–1.40)	0.99 (0.70–1.40)
Obesity, yes vs. no	2.59 (1.95–3.46) ***	2.60 (1.95–3.47) ***	2.60 (1.95–3.46) ***	2.60 (1.95–3.46) ***
Hypertension, yes vs. no	1.17 (0.84–1.64)	1.17 (0.84–1.63)	1.17 (0.84–1.64)	1.17 (0.84–1.63)
Diabetes, yes vs. no	8.48 (6.12–11.76) ***	8.50 (6.14–11.78) ***	8.48 (6.11–11.76) ***	8.48 (6.12–11.75) ***
Cholesterol/HDL-C	0.94 (0.86–1.04)	0.94 (0.86–1.04)	0.94 (0.86–1.04)	0.94 (0.86–1.04)
Hyperuricemia, yes vs. no	1.09 (0.79–1.49)	1.10 (0.80–1.50)	1.09 (0.80–1.50)	1.09 (0.80–1.50)
Cumulative smoking, pack/year	1.00 (0.99–1.01)	1.00 (0.99–1.01)	1.00 (0.99–1.01)	1.00 (0.99–1.01)
Alcohol consumption amount, g/week	1.00 (1.00–1.01)	1.00 (1.00–1.01)	1.00 (1.00–1.01)	1.00 (1.00–1.01)
Betel nut use, ex vs. non	2.41 (1.31–4.44) **			
Betel nut use, current vs. non	2.88 (1.35–6.15) **			
Quantity of betel nut use per day				
≤5 pieces/day vs. none		2.29 (0.85–6.19)		
>5 pieces/day vs. none		2.66 (1.53–4.64) **		
Duration of betel nut use, years				
≤10 years vs. none			2.44 (1.32–4.53) **	
>10 years vs. none			2.80 (1.33–5.90) **	
Cumulative exposure, piece-year ^a^				
≤100 piece-year vs. none				2.24 (1.10–4.58) *
>100 piece-year vs. none				2.88 (1.52–5.47) **

HDL-C, high-density lipoprotein-cholesterol. ^a^ Quantity per day in piece × duration in year; * *p* < 0.05, ** *p* < 0.01, *** *p* < 0.001.

## Data Availability

The data presented in this study are available on request from the corresponding author. The data are not publicly available as the authors were unable to find a proper and valid data repository for the data used in this study.

## Supplementary Materials

The following are available online at https://www.mdpi.com/2072-6643/13/3/914/s1, Table S1: Comparisons of participants’ clinical characteristics between NAFLD subjects with and without betel nut chewing; Table S2: Logistic regression model for risk of significant liver fibrosis (defined as APRI ≥ 0.5).
