# Supplementary material for: A Positive Relationship between Betel Nut Chewing and Significant Liver Fibrosis in NAFLD Subjects, but Not in Non-NAFLD Ones
Source: Nutrients. 2021 Mar 11;13(3):914. doi: 10.3390/nu13030914 (PMC7998430; doi:10.3390/nu13030914)
Supplement: Supplementary file 1 [file nutrients-13-00914-s001.pdf]

Table S1. Comparisons of participants' clinical characteristics between NAFLD subjects with and without betel nut chewing.

Variables	Betel Nut Chewing		*p*-Value
	No (*n* = 1772)	Yes (*n* = 143)	
Age, years	48.47 ± 11.2	45.9 ± 10.8	0.009
Male	1251 (70.6)	141 (98.6)	<0.001
BMI, kg/m^2	26.7 ± 3.1	27.6 ± 3.6	0.004
SBP, mmHg	121.0 ± 15.8	125.5 ± 17.2	0.001
DBP, mmHg	72.2 ± 10.4	75.2 ± 11.4	0.001
FPG, mg/dL	96.6 ± 24.2	100.4 ± 28.7	0.074
ALT, U/L	40.2 ± 28.4	50.6 ± 41.0	0.003
AST, U/L	27.9 ± 16.2	38.9 ± 45.3	0.008
Cholesterol, mg/dL	205.4 ± 36.1	209.2 ± 38.6	0.233
Triglyceride, mg/dL	164.5 ± 96.3	237.7 ± 185.9	<0.001
HDL-C, mg/dL	43.68 ± 10.1	39.1 ± 10.3	<0.001
Cholesterol/HDL-C	4.9 ± 1.8	5.7 ± 1.6	<0.001
Creatinine, mg/dL	0.90 ± 0.22	0.95 ± 0.14	<0.001
Uric acid, mg/dL	6.6 ± 1.4	7.0 ± 1.6	0.005
Hypertension	316 (17.8)	37 (25.9)	0.017
Diabetes mellitus	267 (15.1)	28 (19.6)	0.150
Alcohol consumption, gm/wk	12.5 ± 24.8	34.5 ± 29.9	<0.001
Cumulative smoking, pack-year	4.8 ± 12.9	19.4 ± 17.6	<0.001
Exercise ≥ 3/wk	199 (11.2)	12 (8.4)	0.297
Betel nut chewing, none	1772 (100)	0 (0)	<0.001
Ex-chewing	0 (0)	89 (62.2)	
Current	0 (0)	54 (37.8)	
Duration of betel nut use, years	0 ± 0	10.9 ± 8.2	<0.001
Duration of betel nut use, none	1772 (100)	0 (0)	<0.001
≤10 years	0 (0)	96 (67.1)	
>10 years	0 (0)	47 (32.9)	
Quantity of betel nut use per day	0 ± 0	13.0 ± 9.8	<0.001
Quantity of betel nut use, none	1772 (100)	0 (0)	<0.001
≤5 pieces/day	0 (0)	32 (22.4)	
>5 pieces/day	0 (0)	111 (77.6)	
Cumulative exposure of betel nut, piece-year	0 ± 0	162.0 ± 198.8	<0.001
Cumulative exposure, none	1772 (100)	0 (0)	<0.001
≤100 piece-year	0 (0)	68 (47.6)	
>100 piece-year	0 (0)	75 (52.4)	
APRI score	0.29 ± 0.22	0.42 ± 0.56	0.004
APRI score ≥ 0.5	126 (7.1)	24 (16.9)	<0.001
NFS	−2.87 ± 1.18	−2.53 ± 1.25	0.002
NFS ≥ −1.455	347 (19.6)	42 (29.4)	0.005

Data expressed as mean ± standard deviation or number (percent). NAFLD: non-alcoholic fatty liver disease, BMI: body mass index, SBP: systolic blood pressure, DBP: diastolic blood pressure, FPG: fasting plasma glucose, ALT: alanine aminotransferase, AST: aspartate aminotransferase, HDL-C, high-density lipoprotein-cholesterol, eGFR: estimated glomerular filtration rate, APRI: AST to Platelet Ratio Index, NFS:NAFLD fibrosis score. Normal reference value: FPG: < 100mg/dL, AST: male:10–50 U/L, female:10–35 U/L, ALT: male: < 50 U/L, female: < 35 U/L, Cholesterol: < 200mg/dL, Triglyceride: <150 mg/dL, HDL-C: male: > 40 mg/dL, female: >50 mg/dL, Creatinine: male:0.70–1.20 mg/dL,female:0.50–0.90 mg/dL, Uric acid: male: <7mg/dL, female: < 6mg/dL.

Table S2. Logistic regression model for risk of significant liver fibrosis (defined as APRI ≥0.5).

**Variables**	**Multivariate Analysis** [a]	
	**OR (95% CI)**	***p*-Value**
NAFLD (+), Betel nut chewing (−)	Reference	
NAFLD (−), Betel nut chewing (−)	0.43 (0.30–0.61)	<0.001
NAFLD (−), Betel nut chewing (+)	0.48 (0.19–1.23)	0.127
NAFLD (+), Betel nut chewing (+)	2.37 (1.42–3.95)	0.001

NAFLD: non-alcoholic fatty liver disease, APRI: AST/platelet ratio index, BMI: body mass index, HDL-C: high-density lipoprotein cholesterol, OR: odds ratio, CI: confidence interval. [a] Adjusted for age, sex, BMI, hypertension, diabetes, Cholesterol/HDL-C ≥ 5, Hyperuricemia, cumulative exposure of smoking (pack-year) and alcohol consumption amount (g/week).
